# Water quality assessment of a long-term established extensive green roof under tropical conditions

**DOI:** 10.1007/s10661-026-15093-8

**Published:** 2026-02-23

**Authors:** Renato Castiglia Feitosa, Jaime L. M. Oliveira, Bruno Amorim de Souza

**Affiliations:** 1 Department of Sanitation and Environmental Health, National School of Public Health, Oswaldo Cruz Foundation (Fiocruz), Rio de Janeiro, Brazil; 2 General Coordination of Campus Infrastructure, Oswaldo Cruz Foundation (Fiocruz), Rio de Janeiro, Brazil

**Keywords:** Green roofs, Rainwater, Runoff, Water quality, Sustainability

## Abstract

Green roofs comprise vegetated systems that aim to offset the effects of urbanisation towards more sustainable environmental conditions. While these vegetated systems are a key component of sustainable drainage systems, reducing stormwater runoff volume, their role as a source or sink of pollutants remains debated, particularly in tropical climates where long-term data for mature systems are critically scarce. This study aims to assess the water quality of stormwater runoff from a mature extensive green roof (established for 7 years) in Rio de Janeiro, Brazil, under tropical climate conditions. Two identical (1.8 m^2^) green roof and metallic roof prototypes were monitored over an 11-month period, encompassing 11 rainfall events. Samples of rainwater (RW) and stormwater runoff from the metallic roof (MR) and green roof (GR) were analysed for physicochemical and microbiological parameters, such as colour, pH, turbidity, total dissolved solids (TDS), total organic carbon (TOC), total nitrogen (TN), total coliforms (TC) and *Escherichia coli* (EC). Results indicate that green roof runoff exhibited significantly higher concentrations of colour, TDS, TOC, TN and microbiological contaminants than rainwater and metallic roof stormwater runoff. Given that these findings highlight the role of green roofs in influencing physicochemical and microbiological parameters in rainwater, the overall environmental impact must be weighed against the significant reduction in runoff volume they provide. The mitigation of stormwater runoff by green roofs can offset their role as a source of pollutants in rainwater. However, it must be emphasised that integrated load-based assessments and careful substrate management are needed in tropical urban settings.

## Introduction

Due to increased urbanisation, green areas are being replaced by impervious surfaces, altering land perviousness and thermal properties. To restore urban hydrological functions, sustainable solutions such as green roof systems are increasingly being proposed. Green roofs comprise a multi-layered structure composed of soil and plants, classified as extensive (soil layer up to 150-mm depth) or intensive (soil layer > 150-mm depth) (Kosareo & Ries, 2007; Mentens et al., 2006). The ecological advantages of these vegetated systems comprise the mitigation of air temperature (De Cristo et al., 2025; Rafael et al., 2021; Schmidt et al., 2025), increases in biodiversity (He et al., 2024; Wooster et al., 2022), improvements in air quality (Banirazi Motlagh et al., 2021), and attenuation of stormwater runoff (Breulmann et al., 2025; Kolasa-Więcek & Suszanowicz, 2021; Shafique et al., 2018a; J. Wang et al., 2022; Xu et al., 2025), among others.

Regarding green roof stormwater runoff, quantity is not the only factor to consider; quality is also important. Green roofs can reduce pollutants through absorption and filtration processes, due to their multi-layer composition, which includes vegetation, substrate, filter and drainage system (Liu et al., 2019; Vijayaraghavan & Joshi, 2014). The soil substrate can alter its function, either capturing or leaching substances that can be removed or added to stormwater during the percolation process through the green roof (Gong et al., 2014; Shafique et al., 2018). It is reported that the concentrations of nutrients and the suspended solids released from green roofs are greater than those of conventional roofs’ runoff (Liu et al., 2019), which raises questions about the role of green roofs as a sink for physicochemical and microbiological parameters (Gnecco et al., 2023a, b).

In comparison with conventional roofs, several authors have evaluated the role of green roofs as sinks or sources of physicochemical and microbiological parameters in rainwater runoff (Berndtsson, 2010; He et al., 2024; Marín et al., 2023; Razzaghmanesh et al., 2014; Rowe, 2011; Santana et al., 2022; Shahmohammad et al., 2022; Vijayaraghavan, 2016; Vijayaraghavan & Joshi, 2014; Yan et al., 2024; Zhang et al., 2015). However, a significant gap remains regarding mature green roof studies in the southern hemisphere. Fewer than 5% of published studies combine the variables of tropical climate, shallow soil substrates and long-term establishment (over 5 years) green roof setup. Consequently, this study explicitly evaluates the role of a 7-year-old extensive green roof in a tropical environment on runoff quality, providing essential data on the transferability of green roof performance from temperate to tropical contexts.

## Methodology

### Location and climate characteristics

**Fig. 1:**
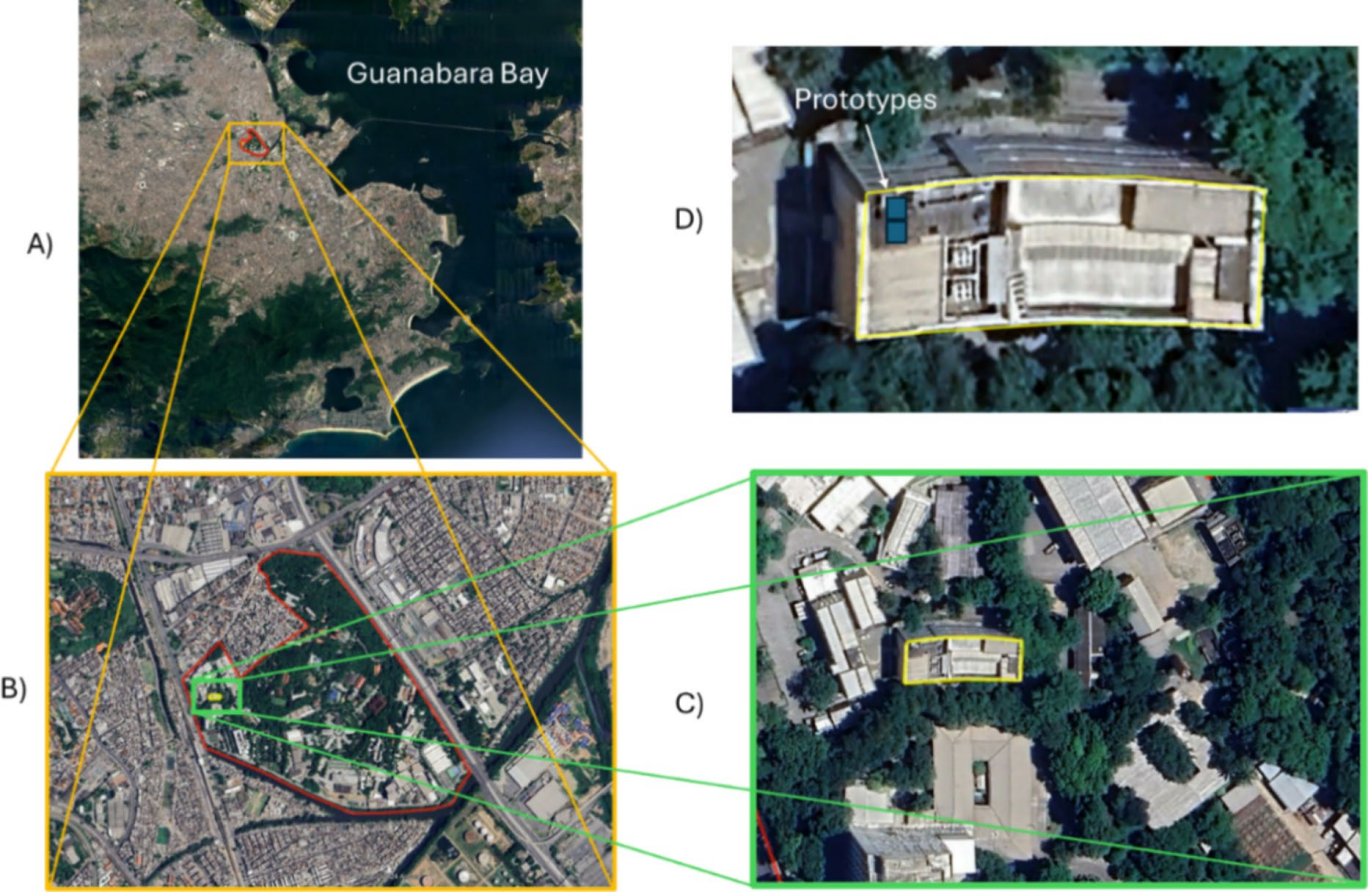
Location of the Oswaldo Cruz Foundation campus and the position of the prototypes. **A** Position of the campus—red polygon. **B** Building location in the campus—green square. **C** Building perimeter—yellow square. **D** Position of the prototypes

The Campus of Oswaldo Cruz Foundation (Fiocruz), located on the north side of the city (679,502 E/7,468,990 S—UTM coordinates), comprises an area of 800,000 m^2^ near Guanabara Bay. It is surrounded by an urban area with heavy traffic, making it susceptible to external particulate matter pollution (Fig. 1). The inner part of the campus comprises a significant proportion of vegetated areas that provide shelter for small mammals and birds, exhibiting notable diversity in an urban setting (Fig. 1C).

Rio de Janeiro is located in southwestern Brazil and has a tropical climate with warm conditions year-round. During the hottest months from December to March, temperatures exceeding 30°C are typical, while in the coolest months, temperatures are mostly higher than 15 °C and can occasionally reach 30 °C (INMET, 2024). The regional rainfall regime varies from a humid season in the warmest months to a dry season in the middle of the year, along with the coolest months. This tropical climate is highly relevant for water quality studies, as high solar radiation and elevated temperatures accelerate chemical reaction rates and the degradation of organic matter within the substrate. Furthermore, the distinct rainfall comprising humid (November to March) and dry (April to October) seasons directly influences the atmospheric deposition of pollutants and the subsequent washing effect on the roofs. 

**Fig. 2:**
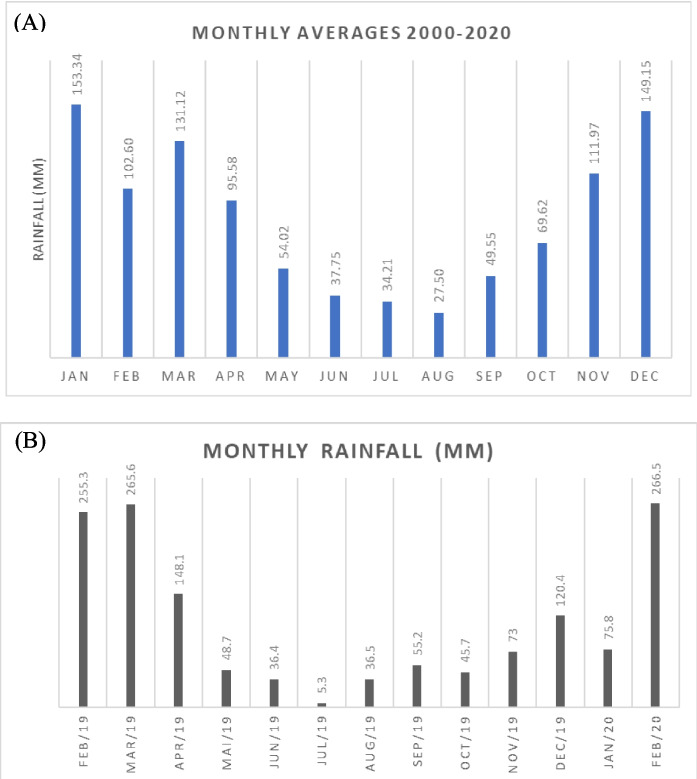
Average monthly rainfall. **A** Monthly averages from 2000 to 2020 from three meteorological stations in the vicinity of the site study. **B** Monthly rainfall from February 19 to February 20, measured by a rain gauge, near the site study

As shown in Fig. 2A, according to rainfall data collected at three meteorological stations in the neighbourhood of the site of the study from 2000 to 2020 (Alerta Rio, 2025), the wettest months occur from November to March (the warmer season), and the driest months occur from April to October (the cooler season). Additionally, a rain gauge was installed 100 m from the site to measure rainfall heights from January 2019 to February 2020 (Fig. 2B). Although this period is significantly shorter than that shown in Fig. 2A, the same trend is observed.

### Experimental setup

The water quality of runoff from a metallic roof (galvanised steel tile) and a green roof was compared with that of direct rainwater. Two scaled-down structures (prototypes), measuring 150 cm in length, 120 cm in width, 100 cm in front height and 120 cm in back height and covered with a metallic roof, were assembled at the top of an existing building in Fiocruz. To ensure equivalence and comparability between the samples, both prototypes were installed with the same slope (approximately 13%), the same orientation and the same total surface area, differing only in the roof covering: one with a conventional galvanised steel tile (MR) and the other with a green roof (GR).

**Fig. 3:**
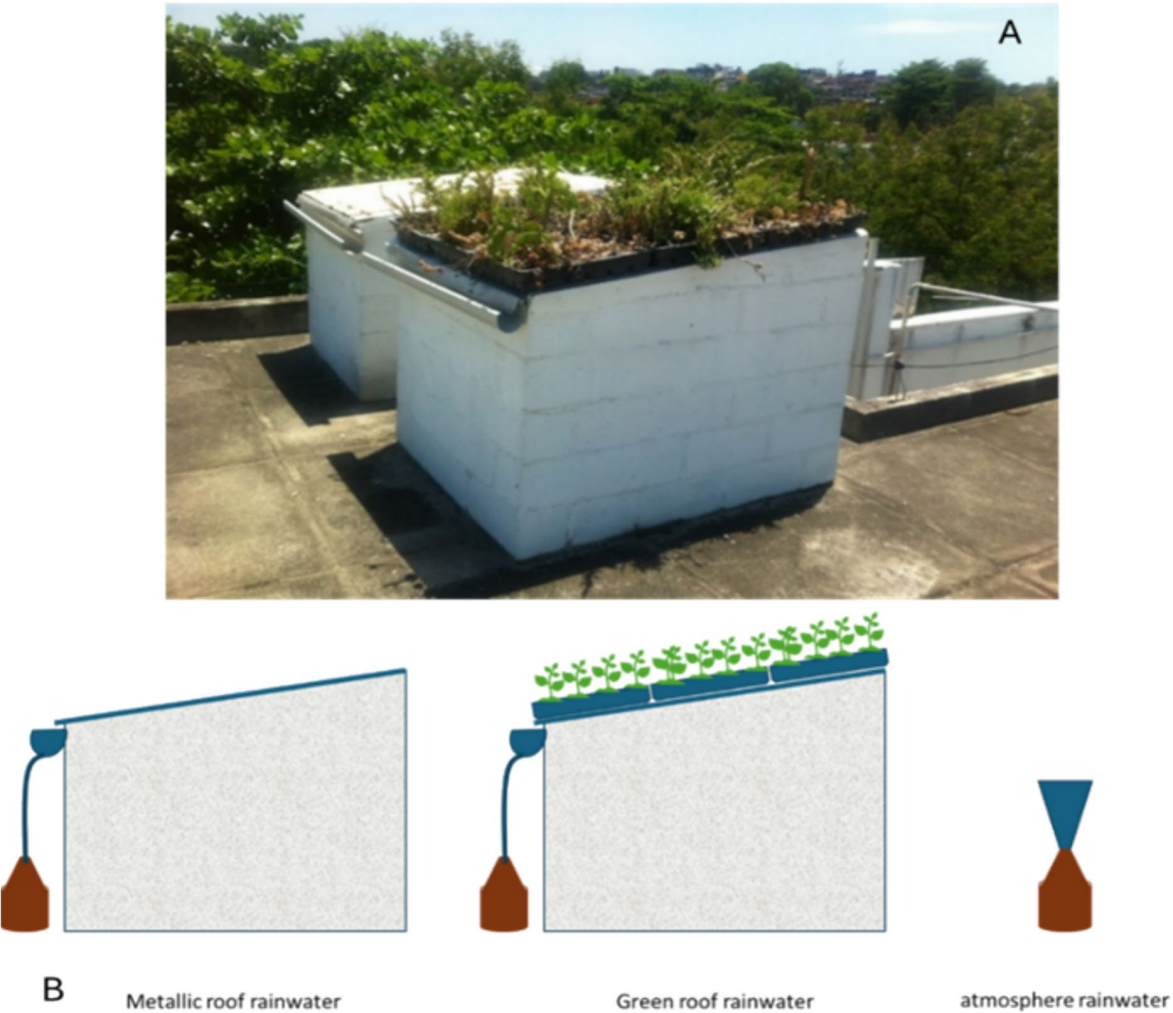
**A** Metallic roof and green roof prototypes. **B** Scheme of rainfall water sampling

As shown in Fig. 3, succulent plants were planted in plastic containers (400 × 500 mm) that comprise a lightweight, modular system with a shallow substrate (5-cm thick). This methodology enables off-site processing across all stages of the green roof installation, including planting, cultivation, plant development and maintenance. The specific 5-cm soil depth was chosen based on the extensive green roof concept, which emphasises low structural loads. This characteristic may be a key factor in applying this system at the city scale, as such loads may not require structural reinforcement. The substrate was characterised as a mixture of sand (50%), silt/clay (25%) and leaf mould (25%), weighing between 60 and 80 kg/m^2^ under both dry and saturated conditions.

The succulent species selected—*Callisia repens*, *Echeveria*, *Crassula ovata*, *Crassula lycopodioides*, *Sedum*, *Tradescantia* and *Pachyveria*—were chosen because they are drought-resistant and exhibit crassulacean acid metabolism (CAM), which protects the photosynthesis process from H_2_O and CO_2_ stresses by allowing the plant to switch between photosynthesis pathways (Black et al., 2003). This choice is contextualised by the need for plants that can survive Rio’s intense heat and sporadic rainfall without artificial irrigation.

A significant aspect of this study is its long-term nature. The experiment was established in 2012 and reached biological equilibrium over 7 years prior to this sampling period. During this time, the system received no fertilisers, relying solely on natural rainfall. This provides a realistic view of vegetation development and substrate stability in a low-maintenance urban setting.

### Sampling protocol and methodological analysis

As shown in Fig. 3, the runoff water samples were collected in 4-L amber glass bottles connected to PVC gutters, which were covered to prevent the deposition of particulate matter between rainfall events, ensuring that the samples were restricted to roof runoff. Atmospheric rainwater was collected in an amber glass bottle through a metallic funnel with a composition similar to that of the metallic roof. Thus, the rainwater samples were obtained from three matrices: rainwater directly from the atmosphere (RW), rainwater runoff from the metallic roof (MR) and rainwater runoff from the green roof (GR). The amber glass bottles used were previously washed with neutral detergent, rinsed with ultrapure water and dried to avoid contamination.

Eleven monthly rainfall samplings were collected between April 2019 and February 2020. Given the lack of July sampling due to the absence of precipitation and the first January sampling having a lower volume than that observed in June, it was decided to conduct an additional January sampling to maintain the planned number of 11 samples.

The experimental location on the top of a building is chosen to avoid rainfall interference from surrounding dwellings. However, the building’s rooftop presented limitations due to structural load capacity and the available space for building replicated prototypes. Thus, although the absence of multiple prototypes for each roof type is a limitation regarding spatial replication, the longitudinal approach (11 monthly samples) provides a robust temporal dataset to compare the matrices (RW, MR and GR).

The samples were analysed for total dissolved solids (TDS), apparent colour, pH, turbidity, total organic carbon (TOC), total nitrogen (TN), total coliforms (TC) and *Escherichia coli* (EC). All analyses were performed according to *Standard Methods for the Examination of Water and Wastewater*—APHA (Baird R. Bridgewater L., 2017). Table 1 presents the methodological analysis used.

**Table 1:** Physicochemical and microbiological methods used for the characterisation of different rainwater samples

Parameter	Method used
TDS	Gravimetric (number 2540 C)
Apparent colour	Spectrophotometric (number 2120 C)
pH	Potentiometric (number 4500B)
Turbidity	Nephelometric (number 2130B)
Total coliform	Enzyme substrate (number 9223B), Colilert
*Escherichia coli*	
TOC	Thermal combustion with infrared detection
TN	Thermal oxidation with chemiluminescence detection

### Data analysis

Data from quantitative analysis of the different rainwater samples (GR, MR and RW) were submitted to the Shapiro–Wilk test for normality evaluation. Afterwards, these samples were compared in pairs across three different scenarios: (1) RW × GR, (2) RW × MR and (3) GR × MR. The parameters analysed in scenarios 1 and 2 were treated as dependent variables, whereas those analysed in scenario 3 were treated as independent variables. For normally distributed parameters, statistical comparisons were conducted to assess whether the means and variances differed significantly using a *t*-test. Conversely, for parameters that did not follow a normal distribution, comparisons were made using the non-parametric Mann–Whitney and Wilcoxon tests. All tests were evaluated at a 95% confidence interval (*p* = 0.05) using Microsoft Excel and the statistical package Past (version 2.17C).

## Results and discussion

**Fig. 4:**
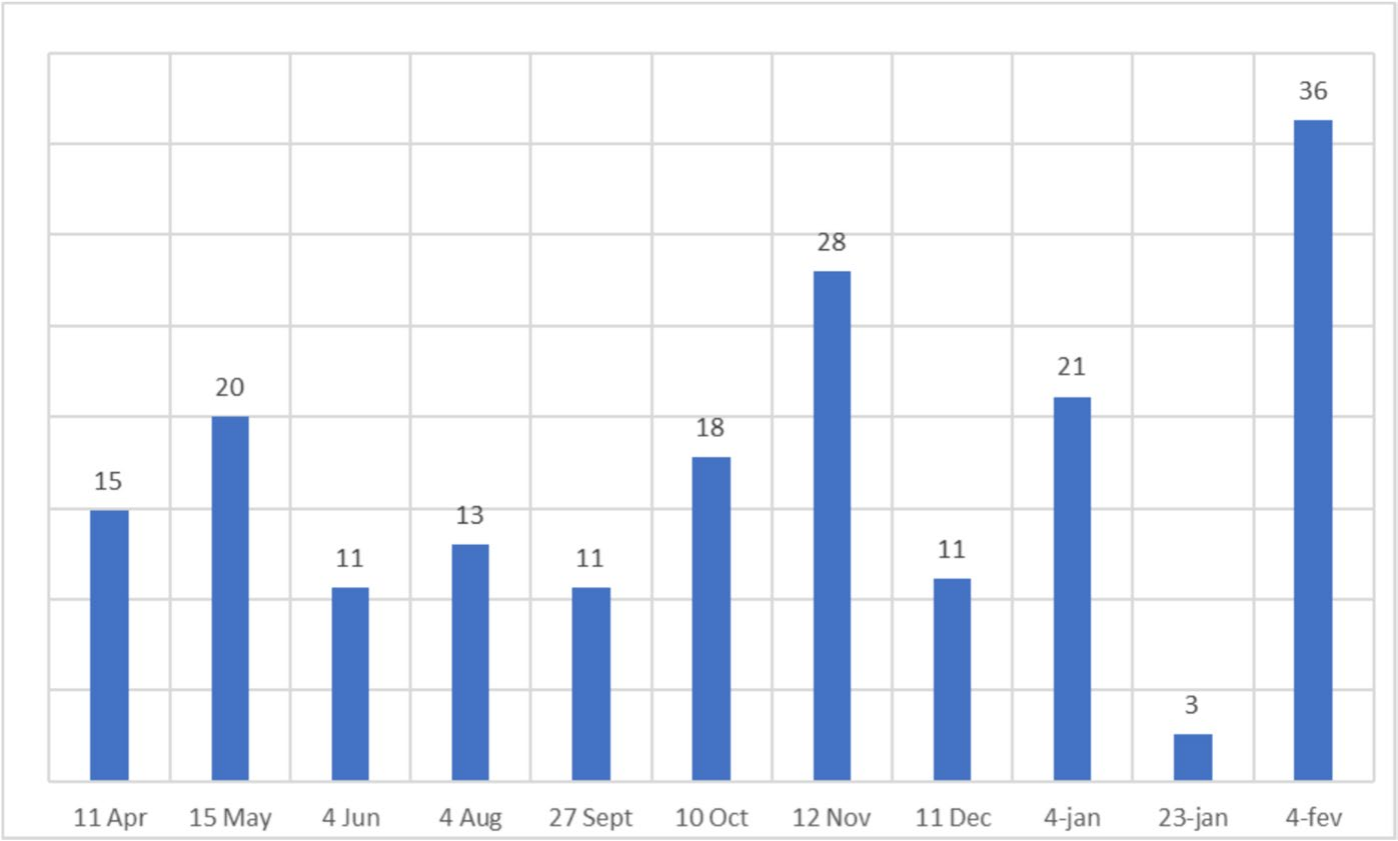
Rainfall (mm) in the 24 h prior to the sampling, from April 2019 to February 2020

The results comprise samplings of 11 rainfall events from April 11, 2019, to February 4, 2020, from the three different systems: MR, GR and RW. The rainfall events were selected because precipitation amounts were sufficiently high, allowing rainwater to percolate through the soil. Figure 4 shows the sampling days and the rainfall height 24 h before sample collection. The sampling period encompasses distinct seasonal transitions typical of a tropical climate. Events from April to June represent the transition from autumn to winter, while the August to October period captures the dry season and early spring. The events from November to February coincide with the warm, rainy summer. As shown in Fig. 4, the highest rainfall intensity (36 mm) occurred in February, during the peak of the wet season.

The water quality analysis is divided into four sections. The first section compares the rainwater quality parameters between the three matrices across 11 rainfall events. The second section provides a statistical analysis of rainwater quality parameters; the third section analyses the relationship between rainwater volumes and concentrations of water quality parameters; and the fourth section assesses green roof runoff water quality against water quality standards.

### Evaluation of physicochemical and microbiological rainwater quality parameters

**Fig. 5:**
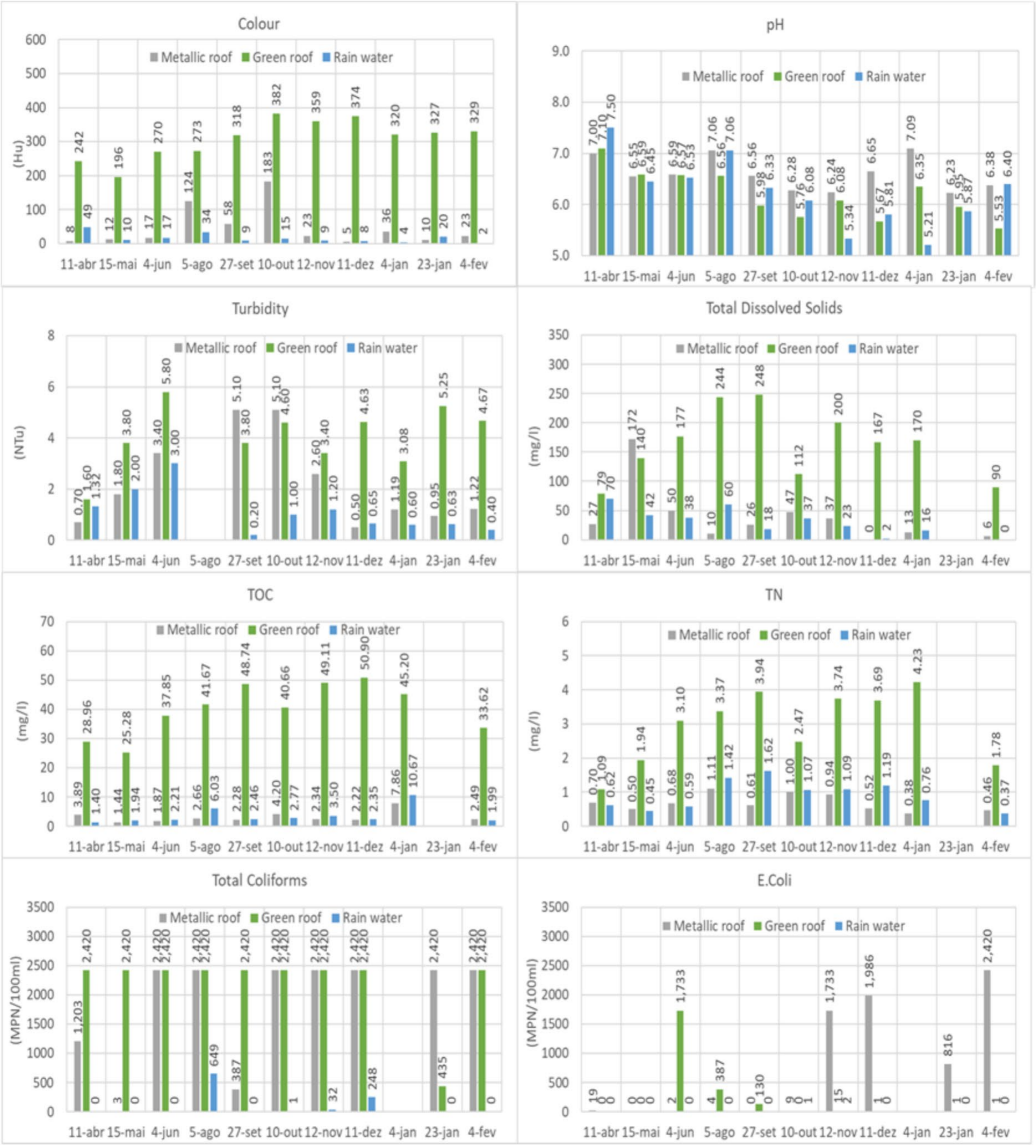
Physicochemical and microbiological parameters of the three different matrices, considering 11 rainfall sampling events

In general, the water from RW, MR and GR presented differences in quality and quantity. As expected, the volume of water collected in GR was lower due to the water retention in the soil and the plants. The water quality of the rainfall matrices was dependent on the material characteristics with which the water came into contact (roof and soil substrate) before the sampling events. Figure 5 presents a comparison of water quality parameters among three different matrices: metallic roof (MR), green roof (GR) and rainwater (RW), based on 11 rainfall sampling events as described in Fig. 4.

#### Colour

The apparent colour levels of the RW varied from 2 to 49 Hu, which were significantly lower than those of MR and GR. However, the RW outliers’ values (34 and 49 Hu) were observed, probably due to particle deposition on the internal funnel’s surface. The highest value occurred in the first sampling, when metallic particles associated with the first use of the funnel used to collect the rainfall may have influenced the colour of the rainwater sample. The second outlier was observed during the dry season, which may be due to the accumulation of atmospheric particles on the funnel’s surface during a prolonged drought preceding the rainfall.

A greenish–yellow tone was characteristic of the rainwater samples from GR. Similar findings have been observed in different works (Razzaghmanesh et al., 2014; Santana et al., 2022; Speak et al., 2014; van Seters et al., 2009; Whittinghill et al., 2016, 2024), indicating the role of organic matter and humic substances degradation in the water colour from green roofs. The mechanism is that adding an organic compound to the substrate provides organic carbon (OC). The composition of this material releases humic and fulvic acids, which have a natural yellowish pigmentation (Whittinghill et al., 2024). The colour levels of the GR runoff (196–382 Hu) were significantly higher than those observed for MR (5–183 Hu). Similar findings were observed in a tropical climate in Recife, Brazil (Santana et al., 2022). The differences between these two matrices were greater during the rainy season, when rainfall levels were significantly higher than in the dry season, corroborating the findings from Whittinghill et al. (2024). A similar study, performed in Manchester City (Speak et al., 2014), observed a similar trend, but with GR presenting colour levels twice as high as MR. The differences between the findings of the present study and Santana et al. (2022) versus (Speak et al., 2014) may lie in climate conditions and local rainfall indices. Tropical climate may favour the degradation of organic plant matter, promoting the formation of humic substances that contribute to higher colour levels.

#### pH

RW presented the greatest variation, ranging from 5.21 to 7.50, and may have a more direct relationship with atmospheric pollutants. Rainwater has a slightly acidic pH of around 5.6 due to the dissolution of atmospheric CO_2_ in raindrops (Bayraktar & Turalioglu, 2005) and atmospheric pollution (Granados Sánchez et al., 2010). Any shift in pH level, depending on the type of pollutants transferred to the water droplets, classifies rainwater as either alkaline or acidic. pH values were lower than 5.6 in the rainfall on November 12 and January 4. Among the samplings, the lowest pH levels were observed in warm seasons. Similarly, a greater frequency of acid rain events during spring and summer was reported by Marín et al. (2001), highlighting the potential to oxidise sulphur and other atmospheric components, thereby lowering pH.

For MR, the pH ranged from 6.23 to 7.09, with a mean of 6.6, indicating a slightly acidic characteristic. Even though, as shown in the following statistical analysis, the MR and RW pH are similar, MR showed higher pH levels in 8 of the 11 samplings. During dry periods, dry deposition of alkaline particles and soil minerals on the roof surface neutralises the acidity of rainfall upon contact, leading to an immediate increase in pH (Mendez et al., 2011). In cases where MR levels are higher than RW, as shown in studies conducted in the Brazilian states of São Paulo and Espírito Santo (Annecchini, 2005; May, 2004), it may occur due to the reaction of rainwater with existing particle deposits and with the roof material, consuming hydrogen cations and lowering the pH of the water (Sosa Echeverría et al., 2023). It is also important to highlight that metallic roofs can leach ions into rainwaters affecting their pH. In general, metallic roofs increase rainwater pH by consuming acidity during corrosion, though the runoff typically remains slightly acidic (pH 6.0–6.5) because its buffering capacity is weaker than that of concrete or clay (Farreny et al., 2011; Mendez et al., 2011). However, exceptions occur when pH decreases due to the accumulation of acidic pollutants (such as sulphur dust, bird droppings or decaying leaves) or secondary chemical reactions (such as hydrolysis) of dissolved metals in storage (Lee et al., 2012; Wallinder et al., 2001).

Regarding GR, the pH varied between 5.53 and 7.10, presenting, on average, a slightly acidic characteristic compared to MR and RW, which may be attributed to the formation of humic substances in the substrate layer (Cristiano et al., 2023). However, it is essential to emphasise that these results contrast with those observed by other authors, who state that the pH of green roofs is higher than the pH of rainwater (Chen et al., 2018; Gnecco et al., 2013a, 2013b; Marín et al., 2023; Qianqian et al., 2019; Santana et al., 2022; Teemusk & Mander, 2007; Todorov et al., 2018; Wang et al., 2017). A crucial point about this divergence is the age of the green roof: The extensive green roof presented in this work has a 5-cm depth and was established in 2012. As pointed out, considering a compilation of studies evaluating the age of green roofs, the soil pH and substrate depth decline over time (Thuring & Dunnett, 2014).

#### Turbidity

In general, the turbidity of all the rainfall matrices was low. Among the 30 samples, only two records (5.80 and 5.25 NTU) for GR were slightly above 5 NTU. As expected, the turbidity of RW was generally lower than that of MR and GR, ranging from 0.20 to 3.00 NTU. Only the samples collected in May and June from RW showed the highest turbidity values (2.00 and 3.00 NTU), likely due to particulate atmospheric deposition in the funnel used to collect RW before the rainfall event.

For MR, turbidity ranged from 0.50 to 5.10 NTU, peaking in September and October, likely due to low rainfall, which can concentrate particulate matter deposited on the roof surface. The GR turbidity varied from 1.60 to 5.80 NTU, which was slightly higher than MR, consistent with other works in the literature (Abuseif, 2023; Park et al., 2022; Vijayaraghavan, 2016). For green roofs, runoff turbidity depends strongly on substrate composition (Morgan et al., 2011) and on dissolved substances and suspended particles (Santana et al., 2022). The turbidity levels reported by Santana et al. (2022) for green roofs were substantially higher, ranging from 56,00 to 743,00 NTU, than those observed in the present work. As previously mentioned, the green roof used in the present study was established in 2012. In contrast, the results presented by Santana et al. (2022) were obtained using a younger green roof system. In addition, the substrate content and composition of the thinner particulate sediments (treated sewage sludge) used by Santana et al. (2022) may have contributed to an increase in the turbidity of rainwater from GR. It is essential to highlight that the present work demonstrates a decrease in turbidity of green roof runoff over time, consistent with previous work (Morgan et al., 2011).

#### Total dissolved solids (TDS)

Total dissolved solids in RW varied between 0 and 70 mg/L, presenting a positive correlation with colour (*r* = 0.89), where the highest values, corresponding to 70 and 38 mg/L, concurred with the highest values observed for colour. The results indicate that TDS levels found in RW were dependent on locational characteristics and other factors, such as the high content of particulate matter, sea spray and pollution (Mohamed et al., 2019). Considering that the experimental setup was located in an urban area, close to a coastal area, and surrounded by highways, RW may contain traces of sodium chloride and both polluted and unpolluted particulate matter. These atmospheric components can significantly influence the contents of TDS in RW (Dharaka & Priyantha, 2024; Mohamed et al., 2019; Ratnayaka et al., 2009).

Similarly, the MR runoff followed a similar trend, but with a slight increase in TDS compared to RW, ranging from 0 to 172 mg/L. Studies conducted in Nigeria also found higher TDS levels in rainwater compared to roofs made of metal (Nicholas & Ukoha, 2023). The existing atmospheric components in RW can contribute to atmospheric deposition onto MR, thereby increasing the TDS concentrations in MR runoff. TDS in roof runoff varies significantly, being affected by roofing materials, microorganisms, dry deposition, frequency of rain events and location (Alja’fari et al., 2022; Nwogu et al., 2024; Tengan & Akoto, 2022).

The TDS concentration in GR ranged from 79 to 248 mg/L, which was significantly higher than in RW and MR, explaining the higher colour levels in these samples. Various authors have also reported higher TDS in green roof runoff than in standard roof runoff (Razzaghmanesh et al., 2014; Vijayaraghavan & Joshi, 2014). The sources of TDS in green roofs include substrate composition, nutrient leaching, vegetation and local pollution sources. The composition of the substrate can influence TDS levels in green roof runoff (Razzaghmanesh et al., 2014; Shahmohammad et al., 2022). The use of *Sedum* plants can significantly reduce TDS levels in green roofs, indicating that green roofs can be a source of TDS, which worsens water quality runoff (Yan et al., 2024).

#### Total organic carbon (TOC)

TOC concentrations in RW varied between 1.40 and 10.67 mg/L, and outliers were observed in August (6.0) and December (10.7), probably due to the presence of animals, since traces of birds’ feathers were found in these samples. For the MR, the TOC ranged from 1.44 to 7.86 mg/L, slightly higher than for the RW due to atmospheric deposition of particles. The existence of TOC in the waters from RW and MR lies in the fact that these waters incorporate organic matter from the atmosphere and particle deposition on the roof. Organic carbon in the atmosphere is transferred to the Earth’s surface through dry and wet deposition (precipitation) (Iavorivska et al., 2016; Ma et al., 2024). Additionally, the combustion of fossil fuels can also contribute to the source of TOC in rainwater (Ma et al., 2024).

As for the runoff from GR, the concentration of TOC was significantly greater than that of the RW and MR, ranging from 25.28 to 50.90 mg/L, due to the decomposition of plant species and humic composites (Cristiano et al., 2023). Similar results were observed in Singapore, showing that the concentration of organic carbon depends on the plant species and the organic content in the substrate (Lim et al., 2021). Higher concentrations of TOC were also observed in the green roofs’ runoff when compared to the asphalt roof surface and rainwater samples, due to existing organic matter in the soil and the decaying vegetation (Berndtsson et al., 2009; Zhang et al., 2015).

#### Total nitrogen (TN)

The levels of TN in RW and MR varied from 0.37 to 1.62 mg/L and 0.38 to 1.11 mg/L, respectively. Similar levels of TN were found in other parts of the world. In a study conducted in China, sampling from rainwater and an asphalt roof yielded TN levels slightly higher, at 2.63 ± 1.47 and 3.03 ± 1.43 mg/L, respectively (Zhang et al., 2015). In Adelaide, Australia, data from 75 rainwater samples show that TN concentrations ranged from 0.049 to 2.518 mg/L (Wilkinson et al., 2006). The presence of TN in rainwater results from atmospheric deposition, which is a significant component of the global nitrogen biogeochemical cycle (Song et al., 2021). Human activities have substantially increased nitrogen production through agricultural activities. Along with fertilisers, vehicular emissions are also a significant source of nitrogen compounds released into the atmosphere (Florêncio et al., 2022; Zeng et al., 2023).

Regarding the GR, TN concentrations were higher than those of RW and MR, ranging from 1.1 to 4.2 mg/L. Similar studies have also revealed higher concentrations of TN in the runoff from green roofs compared to rainfall and control roofs (Hathaway et al., 2008; Moran et al., 2005; Zhang et al., 2015). The concentration of nitrogen in green roofs depends on the type of soil, the use of fertilisers and the age of the green roofs (Berndtsson, 2010). It is essential to note that the TN levels presented are higher but not substantially higher than those of RW and MR, primarily due to the age of the green roof at the time of sampling (7 years). These results concur with findings from various authors who have observed a decrease in TN concentrations in the runoff from green roofs over time (Lim, 2023; Marín et al., 2023). The reduction of TN in green roofs’ runoff over time occurs due to the decrease in the decomposition of organic matter and its leaching from the substrate (Lim, 2023). It is essential to highlight that the results presented refer to a green roof system that has been devoid of any additional fertilisation process since its implementation in 2012, thereby justifying our results. In contrast, the addition of fertilisers indicates an increase in TN levels over time (Marín et al., 2023).

#### Total coliform (TC) and *E. coli* (EC)

In general, coliforms were detected in most MR and GR samples, indicating the accumulation of particulate and faecal matter on the catchment surfaces. This is consistent with the presence of local fauna (birds and small mammals) attracted by the high-density vegetation surrounding the experimental site. Consequently, these catchment surfaces act as interceptors for animal waste and different types of particulate matter, which are subsequently mobilised into the runoff during rainfall events. For RW, TC was detected in 40% of the samples, specifically in August, October, November and December, with concentrations of 649, 1, 32 and 248 MPN/100 mL, respectively. Coincidentally, traces of birds’ feathers were identified in August and December samplings. TC is bacteria commonly found in the intestines of animals and humans. Thus, the presence of TC in RW indicates that the water is exposed to dust, birds and other atmospheric pollutants (Santana et al., 2022). EC was present in 20% of the RW samples but with negligible concentrations (< 2 MPN/100 mL), indicating insignificant levels of faecal contamination.

Regarding the microbiological concentrations of MR, TC was detected in all samplings, with concentrations exceeding 1000 MPN/100 mL, except in May (3.1 MPN/100 mL) and September (387.3 MPN/100 mL). As previously noted, MR is susceptible to dry deposition through atmospheric particulates, which can affect the water quality of the runoff. Similar findings were observed by Tengan and Akoto (2022). According to these authors, the deposition of aerosols, organic matter, leaves, debris, airborne microorganisms and animal stools on rooftops can contribute to elevated total coliform concentrations in roof runoff. EC was detected in 8 out of 10 samples, with negligible concentrations ranging from 2 to 18.9 MPN/100 mL in 4 samples. The concentrations of the four remaining samples ranged from 816.4 to 2416.6 MPN/100 mL, likely due to the high tree density in the vicinity of the building where the experiments were conducted. Contact between excretions from birds and other animals and the roof can be a source of EC. The presence of these microorganisms was also detected in standard roofs (Tengan & Akoto, 2022).

The TC concentrations observed for GR were the highest and positive for all samples. Except for the January sampling (435.2 MPN/100 mL), TC levels were above 2000 MPN/100 mL. Similar results were observed with higher concentrations in New York on a large green roof but with the evident presence of birds (He et al., 2024). In Sweden, the same trend was also observed in the runoff from green roofs (Hussain & Berndtsson, 2012; Santana et al., 2022). The presence of TC in green roofs’ runoff is expected once this coliform group is familiar in the environment (such as soils) and the intestines of animals (Mack, 1977). The presence of EC occurred in 7 out of 10 samples, of which three had significantly lower values (1 MPN/100 mL) than the others (14.6, 129.6, 387.3 and 1732.9 MPN/100 mL). These microorganisms can be found in the intestines of warm-blooded animals such as mammals and birds (Basavaraju & Gunashree, 2022). Thus, contamination of rainwater from green roof runoff must be associated with the presence of animals on these roofs. A study conducted in New York City found significant concentrations of EC in all green roof runoff samples (He et al., 2024). These authors associated the presence of these microorganisms with colonies of herring gulls inhabiting this vegetated roof. The presence of EC in bird faces was also evidenced in bird stools (Trawińska et al., 2016). Another study performed in Recife (Brazil) also revealed the occurrence of EC in a green roof runoff (Santana et al., 2022), concurring with the results presented, indicating that green roofs can be a source of pathogenic microorganisms.

### Statistical analysis of the rainwater quality parameters

**Fig. 6:**
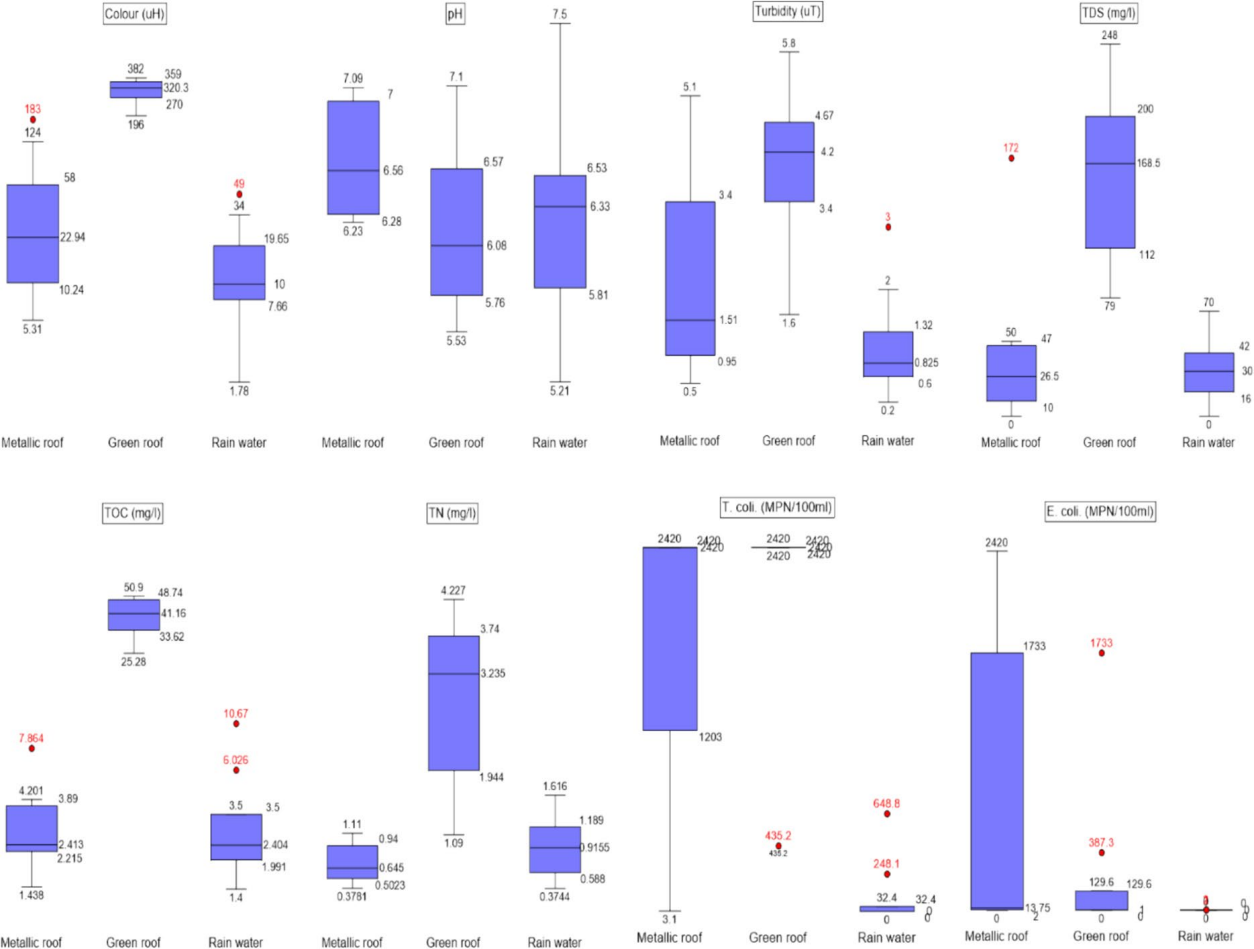
Mean values of the physicochemical and microbiological parameters of three rainwater matrices

Figure 6 presents boxplots of physicochemical and microbiological parameters for the rainwater matrices (RW, MR and GR) based on the 11 sampling events conducted in the present work. There were no significant differences in pH for the three rainwater matrices. Except for EC, the remaining parameters were higher in GR, indicating that green roofs increase the concentration of microbiological and physicochemical parameters in rainwater runoff.

As shown in Table 2, a statistical analysis is presented to evaluate whether the rainwater matrices differ significantly. The normality tests of the rainwater (RW), metallic roof (MR) and green roof (GR) water quality indicated that pH, turbidity and total nitrogen (TN) followed a normal distribution. In contrast, the remaining parameters showed non-normal distributions. Based on this, a two-by-two water quality comparison was performed among the three matrices: MR versus RW, RW versus GR and MR versus GR.

**Table 2:** Statistical analysis of the water quality between rainwater (RW) and metallic roof (MR). *p* values lower than 0.05, highlighted in bold, identify the condition of statistical difference

Parameters	*p* values		
	MR vs. RW	GR vs. RW	MR vs. GR
Apparent colour	**0.03**	**0.03**	**0.001**
pH	0.08	0.82	**0.03**
Turbidity	0.08	**0.00005**	**0.01**
Total dissolved solids (TDS)	0.445	**0.005**	**0.005**
Total organic carbon (TOC)	0.97	**0.005**	**0.003**
Total nitrogen (TN)	0.09	**0.00003**	**0.00006**
Total coliform (TC)	**0.003**	**0.005**	0.41
*E. coli*	**0.01**	**0.03**	0.36

#### MR versus RW

Considering the *p* values shown in Table 3 regarding the comparative analysis between runoff waters from MR and RW, only the apparent colour and microbiological parameters were statistically different (*p* values < 0.05). As far as colour is concerned, even though MR is not relevant in altering runoff colour (Mendez et al., 2011), this parameter can be influenced by the presence of natural organic matter originating from debris and leaves on the roof surface (Köse-Mutlu, 2021). Significant differences in microbiological water quality between rainwater and roof runoff were also observed in other studies (Alja’fari et al., 2022; He et al., 2024; Nwogu et al., 2024; Santana et al., 2022).

**Table 3:**
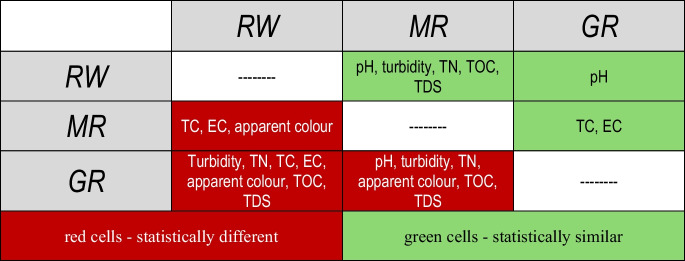
Statistical similarities and differences of the analysed parameters

As for the remaining parameters, no statistical difference was observed in pH levels between rainwater and roof runoff (Nicholas & Ukoha, 2023; Nwogu et al., 2024). Additionally, the turbidity and TDS levels between rainwater and the aluminium roof did not vary significantly (Tengan & Akoto, 2022). Similar findings were observed for TN (Wilkinson et al., 2006; Zhang et al., 2015) and for TOC (Ma et al., 2024).

#### GR versus RW

According to the *p* values presented in Table 3, only pH did not present a statistically significant difference between GR and RW. It is worth noting that this finding contradicts other studies (Chen et al., 2018; Gnecco et al., 2013a, 2013b; Marín et al., 2023; Park et al., 2022; Qianqian et al., 2019; Santana et al., 2022; Teemusk & Mander, 2007; Todorov et al., 2018; X. Wang et al., 2017). However, as previously discussed, these statistical differences can become negligible because soil pH in green roofs declines over time (Thuring & Dunnett, 2014). As far as the remaining parameters are concerned, other authors found similar results to the present study, indicating that green roofs behave as a source of substances that alter the rainwater quality, distinguishing these water quality parameters (Berndtsson et al., 2009; Lim et al., 2021; Razzaghmanesh et al., 2014; Santana et al., 2022).

#### MR versus GR

As shown in Table 3, only the microbiological parameters did not differ between MR and GR. However, the presence of TC and *E. coli* in MR is attributed to the deposition of faecal matter from birds and other animals, which contributes to the higher concentrations of these microorganisms in roof runoff (Tengan & Akoto, 2022). Similar evidence was observed for *E. coli* in green roofs, attributed to the presence of birds (He et al., 2024) and the common presence of TC in soils (Mack, 1977). As for the remaining parameters, GR behaved as a source of physicochemical parameters (Marín et al., 2023) that depend on the soil substrate, whose composition is basically composed of inorganic/mineral materials (80–90%) and organic matter (10–20%) and rainfall characteristics (Hathaway et al., 2008).

#### Summary of the statistical analysis

Table 3 presents a summary of the statistical analyses (*p* < 0.05) of the physicochemical and microbiological parameters among MR, GR and RW. The green cells indicate statistical similarities, whereas the red cells indicate the differences. Regarding rainwater, MR increased only the colour and microbiological concentrations, whereas GR, except for pH, significantly altered the quality of rainwater runoff. In addition, compared with MR, the concentrations of the remaining parameters were higher in green roof runoff, except for the microbiological parameters.

Similar findings also suggest that, depending on the experimental conditions, the runoff of green roofs can contribute to increasing the concentrations of organic matter and nutrients, as well as coliforms and *E. coli* levels (Lim et al., 2021; Marín et al., 2023; Park et al., 2022; Razzaghmanesh et al., 2014; Santana et al., 2022) and microbiological (He et al., 2024; Santana et al., 2022; Tengan & Akoto, 2022). It is essential to note that, despite 8 years of experimentation (2012–2020), the contamination of runoff water from the green roof remained evident.

### Analysis of rainfall heights and the concentration of physicochemical and microbiological parameters

Table 4 presents the Pearson correlation coefficients (*r*), ranging from negligible to moderate, for the relationship between observed concentrations and 24 h of accumulated precipitation (Fig. 4) across three matrices: metallic roof, green roof and rainwater. The analysis of Pearson correlation coefficients (*r*) revealed distinct hydrological behaviour, specifically dilution and washout effects, depending on the water matrix and parameter type.

**Table 4:** Pearson correlation (*r*) between observed concentrations of physicochemical and microbiological parameters and precipitation

Parameter	Metallic roof	Green roof	Rainwater
Apparent colour	−0.019	0.103	−0.404
pH	−0.132	−0.234	−0.175
Turbidity	−0.082	−0.233	−0.125
Total dissolved solids (TDS)	0.006	−0.422	−0.397
Total organic carbon (TOC)	0.093	−0.206	0.049
Total nitrogen (TN)	−0.182	−0.264	−0.497
*T. coli*	0.065	0.505	−0.177
*E. coli*	0.538	−0.255	0.413

Regarding physicochemical parameters, the results predominantly indicate an inverse correlation, suggesting a dilution effect as precipitation volume increases. This trend is particularly evident for total nitrogen (T.N.) and total dissolved solids (TDS). In the rainwater samples, T.N. showed a moderate negative correlation (*r* = −0.497), followed by the green roof runoff (r = −0.264). Similarly, TDS concentrations exhibited moderate negative correlations for both the green roof (*r* = −0.422) and rainwater (*r* = −0.397). These findings align with observations by Zhang et al. (2015), who noted that higher rainfall volumes effectively dilute the solute load accumulated in the atmosphere and on urban surfaces. Other parameters, such as turbidity, TOC and pH, presented weak to negligible negative correlations (|*r*|< 0.25), suggesting their concentrations are less sensitive to rainfall volume variations in this study. The lowest pH levels were observed during the warm seasons, likely due to increased solar radiation, which oxidises atmospheric components such as sulphur, lowering the pH of precipitation (Marín et al., 2001).

Regarding colour, a significant disparity was observed between the green roof and direct rainwater. Rainwater exhibited a moderate negative correlation (*r* = −0.404), indicating a clear dilution of atmospheric aerosols and dust. In contrast, the green roof showed a weak positive correlation (*r* = 0.103). Although weak, the reversal of the sign suggests that intense precipitation events may promote the leaching of organic matter, such as humic and fulvic acids, from the soil substrate into the runoff (Berndtsson et al., 2009). This process counteracts the dilution effect observed in direct precipitation. Conversely, the metallic roof showed a negligible correlation (*r* = −0.019), supporting the view that smooth, inert surfaces do not contribute additional organic staining to the runoff (Mendez et al., 2011).

Microbiological parameters exhibited divergent behaviours depending on the matrix. *E. coli* concentrations showed moderate positive correlations on the metallic roof (*r* = 0.538) and in rainwater (*r* = 0.413). This indicates a washout effect, where increased rainfall volume mobilises accumulated contaminants—likely from atmospheric deposition or avian activity—from smooth surfaces and the air (Mendez et al., 2011).

In contrast, the green roof displayed a distinct functional duality. While it showed a negative correlation for *E. coli* (*r* = −0.255), suggesting the capacity to retain faecal indicators within the substrate or dilute external inputs, it exhibited a significant, moderate positive correlation for total coliforms (*r* = 0.505). This suggests that while the green roof substrate may act as a filter for external pathogens like *E. coli*, intense rainfall events actively mobilise soil-indigenous bacteria (total coliforms) from the vegetation and substrate layers, as previously documented by Hathaway et al. (2008).

### Green roof runoff evaluation according to water quality standards

It is important to highlight that, even though there is no specific federal regulation exclusively for green roof runoff in Brazil, it is regulated according to Brazilian environmental standards (CONAMA 357/2005 for water bodies (class 2) and NBR 15527 (2019) for non-potable reuse). Table 5 evaluates GR runoff according to these environmental standards.

**Table 5:** Comparison between green roof water quality parameters and Brazilian environmental standards

Parameters	Green roof	CONAMA 357 (class2)/NBR15527	Significance
Apparent colour	196.5–382.5	≤ 75 (CONAMA)/15 (NBR)	Very high—exceeds the limit by up to 5×. This indicates high leaching of organic matter (tannins/humic acids) from the substrate
pH	5.53–7.10	6.0–9.0	High—several samples (e.g. 5.53, 5.67) fall below the minimum limit of 6.0, indicating acidification
Turbidity	1.60–5.80	≤ 100 (CONAMA)/2.0 (NBR)	Moderate—complies with river standards but often exceeds the 2.0 NTU limit for non-potable reuse
Total dissolved solids (TDS)	79–248	≤ 500	Low—while it increases relative to rainwater, it remains well within the 500 mg/L limit for water bodies
Total organic carbon (TOC)	25.3–50.9	No direct limit	High—this represents a massive increase compared to the levels in metallic roofs (~2 mg/L). High TOC implies high oxygen demand (BOD), potentially depleting oxygen in receiving waters
Total nitrogen (TN)	1.09–4.23	≤ 2.18 (lotic)/1.27 (lentic)	High—most samples exceed the 2.18 mg/L limit for rivers, indicating the roof acts as a source of nutrient pollution (eutrophication risk)
*E. coli*	Up to 1732.9	≤ 800 (CONAMA)/0.0 (NBR)	Low—only one occurrence above the 800 MPN/100 mL limit for class 2 waters but violates the total absence requirement for non-potable reuse in urban buildings (NBR 15527)

The increase in physicochemical and microbiological parameters from GR runoff is environmentally significant when compared to Brazilian regulatory standards for receiving water bodies, representing a challenge for both urban drainage management and direct discharge into water bodies. Although green roofs can reduce rainfall volume, the results showed that they can act as a point source of nutrients and organic matter during the substrate stabilisation phase. The increases are significant enough to require pretreatment, such as first-flush diversion or filtration, if the water is intended for reuse or if it is discharged into sensitive, low-flow urban streams. However, it is essential to emphasise that these impacts are often punctual and localised. In a practical urban drainage context, the environmental impact of these concentrations is significantly mitigated by dilution. Since green roofs typically represent only a fraction of a building’s total catchment or an urban area’s surface, their runoff is mixed with substantially larger and cleaner volumes of water from inert surfaces (such as metallic roofs) and direct rainfall. This volumetric mixing lowers the final nutrient and organic matter concentrations, bringing the total effluent closer to the assimilation capacity of receiving water bodies and reducing the localised impact on urban stream quality.

The present findings were compared with a similar study conducted in a tropical region of Brazil (Recife, Pernambuco) that evaluated only the Brazilian regulation NBR 15527 (2019). When evaluated against this specific regulation for non-potable reuse, both the present study and Santana et al. (2022) revealed critical non-compliance issues, though the driving factors differed significantly for physicochemical parameters. Regarding microbiological safety, the studies are similar: Both confirmed that green roof runoff fails to meet the strict NBR 15527 (2019) requirement for the complete absence of *E. coli*, identifying these systems as sources of faecal contamination that require disinfection prior to reuse. However, a major divergence occurs in turbidity levels. While the present study recorded values (1.6–5.8 NTU) that frequently exceeded the NBR limit of 2.0 NTU, they were substantially lower than the critical levels reported by Santana et al. (2022), which ranged from 56 to 743 NTU. This discrepancy highlights how substrate composition, specifically the use of treated sewage sludge in Santana’s work, can exacerbate non-compliance, whereas the lower turbidity observed in the stabilised substrate of the present study indicates greater compliance. Additionally, regarding pH, the present study identified instances of acidification (pH < 6.0) violating the NBR minimum limit due to substrate ageing, whereas Santana et al. (2022) typically observed higher pH values characteristic of younger, acid-neutralising substrates.

### Considerations and recommendations

Despite providing a characterisation of rainwater and runoff through physicochemical and microbiological indicators, this study has limitations regarding the scope of the analysed parameters. Recent literature emphasises that water quality assessments in green and conventional roofs benefit significantly from the inclusion of electrical conductivity, total suspended solids (TSS) and inorganic nutrients such as nitrite, nitrate and phosphate (Berndtsson, 2010; Gregoire & Clausen, 2011). Green roofs often act as sources of pollutants rather than sinks, particularly of inorganic nutrients, electrical conductivity and suspended solids. Studies by Berndtsson (2010) and Gregoire and Clausen (2011) indicate that while these systems reduce the total mass of pollutants through water retention, the concentration of nitrogen and phosphorus in the runoff typically exceeds that of conventional roofs due to fertiliser leaching and organic matter decomposition. Furthermore, elevated electrical conductivity reflects the release of dissolved ions from the substrate, while total suspended solids (TSS) levels, though improving with vegetation density, remain susceptible to internal erosion during intense rainfall events. Notably, the high mobility of nitrate resulting from nitrification often leads to its leaching into urban drainage systems when not fully assimilated by plants or the soil matrix.

Furthermore, monitoring heavy metals (e.g. Zn, Cu and Pb) is crucial for evaluating the leaching potential of roofing materials and the impacts of atmospheric deposition in urban environments (Vijayaraghavan et al., 2012). However, according to these authors, green roofs predominantly act as sinks for heavy metals in rainwater. The research demonstrated that the concentrations of metals such as copper, cadmium, lead and zinc in the runoff were significantly lower than those measured in bulk precipitation, indicating the system’s effectiveness in filtering and retaining atmospheric pollutants. However, the authors also noted that the substrate composition is a critical factor, as certain growing media might initially leach specific elements, although the overall net effect tends towards the attenuation of heavy metal loads in urban environments.

The absence of these variables limits the comprehensiveness and international comparability of the results. Therefore, future research should incorporate these parameters to enable a more holistic understanding of the biochemical processes within the substrate and to align the findings with broader international benchmarks for urban runoff quality.

## Conclusions

In addition to their well-documented ability to attenuate stormwater runoff, green roofs significantly alter rainwater quality. This study addresses a critical gap in the literature by evaluating a long-established (7-year-old) extensive green roof under tropical conditions, a context where data on mature systems is scarce. Most existing studies are conducted on recently installed green roofs, which can affect water quality results due to excessive nutrient leaching, potentially deviating the results from a mid- to long-term perspective.

Results revealed that, even after 7 years of establishment and without fertilisation, the green roof acted as a source of microbiological and physicochemical parameters rather than a sink. Statistical analysis revealed a progressive increase in water quality parameters from rainwater to the metallic roof and, most significantly, from the metallic roof to the green roof. While the metallic roof primarily contributed to increases in colour and coliforms, the green roof runoff additionally increased the concentrations of turbidity, total nitrogen (TN), total organic carbon (TOC) and total dissolved solids (TDS).

Compared with metallic roof runoff, green roof runoff showed higher concentrations of turbidity, total nitrogen, colour, total organic carbon and total dissolved solids, indicating that green roofs are a source of these physicochemical parameters. These findings suggest that in tropical climates, the combination of high temperatures and intense rainfall may promote the continuous degradation of organic matter in the substrate, thereby maintaining the roof’s source behaviour over many years. From a practical standpoint for urban drainage management, these results imply that green roofs in tropical sustainable drainage systems must be carefully designed to prevent secondary degradation of water quality. Despite these contributions, this study has limitations, including the lack of physical replication (single prototypes) and the absence of heavy metal and phosphate analysis, which should be addressed in future research to provide a more comprehensive characterisation. To mitigate the observed negative effects on water quality, it is recommended that tropical green roof designs limit organic matter content to 10–15%, incorporate adsorbent materials, such as biochar or zeolites, to enhance nutrient retention, and utilise stable mineral aggregates (expanded clay, pumice and zeolite) to minimise the leaching of solids and turbidity.

## Data Availability

The data that support the findings of this study are available upon request.
